# *Shigella* IpaH0722 E3 Ubiquitin Ligase Effector Targets TRAF2 to Inhibit PKC–NF-κB Activity in Invaded Epithelial Cells

**DOI:** 10.1371/journal.ppat.1003409

**Published:** 2013-06-06

**Authors:** Hiroshi Ashida, Hiroyasu Nakano, Chihiro Sasakawa

**Affiliations:** 1 Division of Bacterial Infection Biology, Institute of Medical Science, University of Tokyo, Shirokanedai, Minato-ku, Tokyo, Japan; 2 Department of Immunology, Juntendo University School Graduate School of Medicine, Hongo, Bunkyo-ku, Tokyo, Japan; 3 Nippon Institute for Biological Science, Shinmachi, Ome, Tokyo, Japan; 4 Medical Mycology Research Center, Chiba University, Inohana, Chuo-ku, Chiba, Japan; Collège de France, France

## Abstract

NF-κB plays a central role in modulating innate immune responses to bacterial infections. Therefore, many bacterial pathogens deploy multiple mechanisms to counteract NF-κB activation. The invasion of and subsequent replication of *Shigella* within epithelial cells is recognized by various pathogen recognition receptors as pathogen-associated molecular patterns. These receptors trigger innate defense mechanisms via the activation of the NF-κB signaling pathway. Here, we show the inhibition of the NF-κB activation by the delivery of the IpaH E3 ubiquitin ligase family member IpaH0722 using *Shigella's* type III secretion system. IpaH0722 dampens the acute inflammatory response by preferentially inhibiting the PKC-mediated activation of NF-κB by ubiquitinating TRAF2, a molecule downstream of PKC, and by promoting its proteasome-dependent degradation.

## Author Summary

In response to bacterial infection, host cells induce a plethora of innate immune responses to combat the infection. However, many bacterial pathogens have developed sophisticated mechanisms to evade the host's immune system. Because NF-κB is crucial for innate immune responses against bacterial infection, bacterial pathogens deploy multiple countermeasures to inhibit NF-κB activation. The invasion and replication of *Shigella* within host cells results in cellular damage and the production of bacterial components that trigger NF-κB activation. Here, we show that the *Shigella* type III secretion system (T3SS) effector IpaH0722, a member of the IpaH E3 ubiquitin ligase family, inhibits NF-κB activation during *Shigella* infection. IpaH0722 preferentially targets the PKC–NF-κB pathway, which is activated in response to danger signals caused by disruption of the phagosomal membrane during the dissemination of *Shigella* into the cytoplasm. IpaH0722 inhibits NF-κB activation by targeting TRAF2, which lies downstream of PKC, for ubiquitination and proteasome-dependent degradation.

## Introduction

The intestinal epithelium deploys multiple sensors and defense systems against microbial intrusion. Microbial components and infection-associated cellular damage are recognized as pathogen-associated molecular patterns (PAMPs) and as danger-associated molecular patterns (DAMPs), respectively. Pattern recognition receptors (PRRs) recognize PAMPs and DAMPs, thus activating the immune system to clear the bacteria and initiate the repair of the injured epithelial lining [1], [2]. Nevertheless, many bacterial pathogens such as *Shigella*, *Salmonella*, *Yersinia*, enteropathogenic *Escherichia coli* (EPEC), and enterohemorrhagic *E. coli* (EHEC), are able to efficiently colonize the intestinal epithelium by utilizing highly evolved mechanisms to counteract host innate defense mechanisms [3]. Previous studies have reported that enteric bacterial pathogens possess distinctive mechanisms to attenuate host inflammatory responses, which is a prerequisite for promoting intracellular and extracellular bacterial survival [4], [5]. For example, during the invasion of the intestinal epithelium by *Shigella*, the Toll-like receptors (TLRs) and Nod-like receptors (NLRs) are the PRRs that recognize PAMPs and DAMPs [6]–[9]. These receptors stimulate host inflammatory signaling pathways, including nuclear factor κB (NF-κB) and mitogen activated protein kinases (MAPK), which culminate in the expression of proinflammatory chemokines and cytokines [6]–[9]. *Shigella* counteract innate immune responses by delivering effector molecules using its type III secretion system (T3SS) [8]–[10]. The targeting molecules and mechanisms of inhibition of the NF-κB pathway are specific to each of the effectors, and *Shigella* are known to deliver OspG [11], OspI [12], OspZ [13], and IpaH9.8 [14]–[17], to efficiently attenuate NF-κB activation thus allowing replication within the intestinal epithelium [8], [9].

IpaH9.8 belongs to IpaH E3 ubiquitin ligase family [16] and it is also called novel E3 ligase (NEL) [18]. The *ipaH* genes, which are originally identified in *Shigella* large virulence plasmid, are conserved by Gram-negative bacterial pathogens, including *Shigella*, *Salmonella*, *Yersinia*, *Edwardshiella ictluri*, *Bradyrhizobium japonica*, *Rhisobium* sp. strain NGR234, *Pseudomonas putida*, *P. entomophila*, *P. fluorescens*, and *P. syringae* [16], [19], [20]. The IpaH family proteins share common structural and functional properties; they contain an N-terminus leucine rich repeat (LRR) and a highly conserved C-terminal region (CTR) [21], [22]. Within the conserved CTR there is a Cys residue that is critical for its E3 ubiquitin ligase activity [16]. Each of the IpaH family effectors, including IpaH9.8 (*Shigella*), SlrP (*Salmonella*), SspH1 (*Salmonella*), SspH2 (*Salmonella*), YopM (*Yersinia*), Y4fR (*Rizobium*), and BIpM (*Yersinia*), has been shown to target specific host proteins in a variety of cell types; some of them act as effectors that attenuate host inflammatory responses, while others modulate host defense responses in plants [17], [23]–[25]. The existence of multiple effectors with E3 ligase activity suggests that a divergent array of E3 ligases is required for promoting bacterial infection and antagonizing host innate defense responses.

The *Shigella flexneri* strain, YSH6000, has three *ipaH* genes (*ipaH9.8*, *ipaH7.8*, and *ipaH4.5*) on its large virulence plasmid and seven *ipaH* genes on its chromosome (*ipaH0722*, *ipaH0887*, *ipaH1383*, *ipaH1880*, *ipaH2022*, *ipaH2202*, and *ipaH2610*) [26]–[28]. We previously showed that all of these IpaH effector proteins are secreted via the T3SS [28], [29], suggesting they have the potential to act as E3 ubiquitin ligase effectors during infection, although their exact roles and host targets during infection, with the exception of IpaH9.8, remain largely unclear [17]. In the mouse lung infection model, we showed that mice infected with the *ΔipaH*-chromosome mutant, which lacks all seven chromosomal *ipaH* genes, had a more severe inflammatory response and less colonizing bacteria compared to the WT strain [28], suggesting that modulation of the host inflammatory response by chromosomal IpaH proteins promotes bacterial infection. Therefore, to gain further insight into the strategies employed by *Shigella* to counteract host innate defence systems during infection, we sought to characterize the chromosomally encoded IpaH effectors. We found that IpaH0722 targets TRAF2 and plays an important role in dampening the PKC–NF-κB pathway, in response to membrane damage generated during *Shigella* invasion of epithelial cells.

## Results

### IpaH0722 dampens NF-κB activation during *Shigella* infection

**Figure 1:**
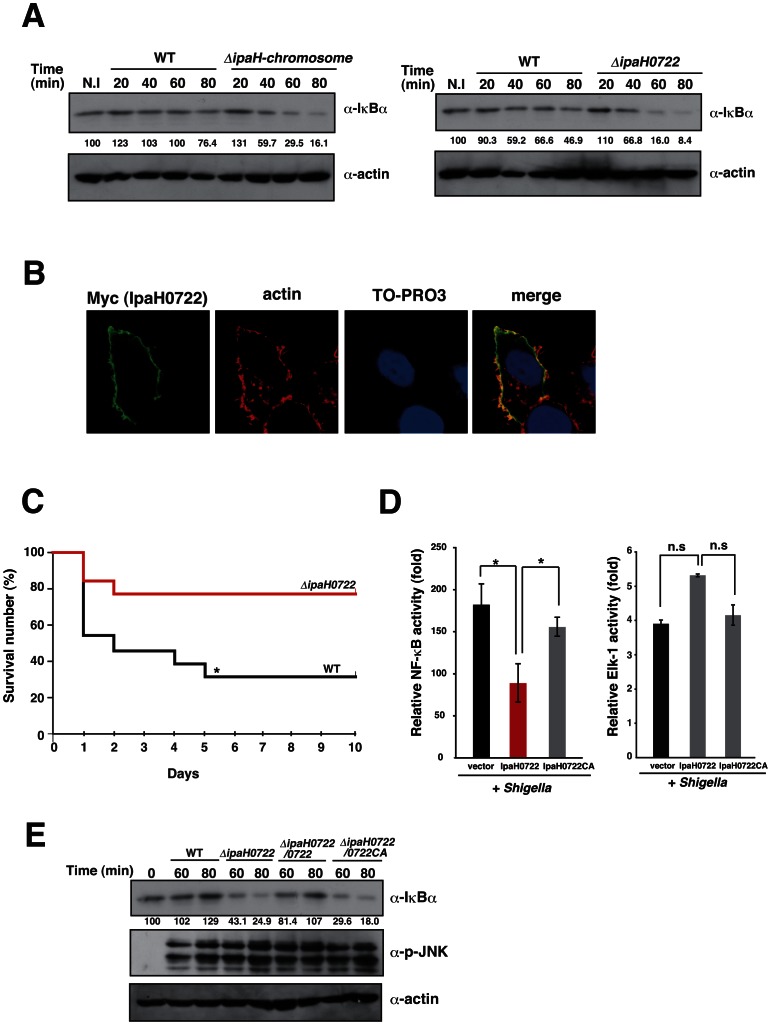
IpaH0722 inhibits *Shigella*-induced NF-κB activation. (A) HeLa cells were infected with *Shigella* WT, *ΔipaH-chromosome* (left), or *ΔipaH0722* (right). The cell lysates were prepared at the indicated time points and subjected to immunoblottings with anti-IκBα. Anti-actin antibody was used as a loading control. The values indicated below the images are the relative intensities of the bands. (B) IpaH0722 localizes to the host cell plasma membrane. Cos-7 cells were transfected with a Myc-IpaH0722 expression vector and immunostained with anti-Myc, actin, and TO-PRO3. (C) The murine pulmonary model of *Shigella* infection. Mice were intranasally inoculated with *Shigella* WT or *ΔipaH0722* (*n* = 13) at 5×10^7^ cfu and survival was recorded for 10 days post-infection. *P<0.05. (D) NF-κB-luciferase and Elk-1-luciferase reporter assays were performed after *Shigella* infection of 293T cells that were transiently transfected with empty vector, IpaH0722, or IpaH0722CA expressing plasmids. Results are presented as ‘fold’ relative to the activity of uninfected or unstimulated cells. *P<0.01, n.s., not significant. (E) HeLa cells were infected with *Shigella* WT, *ΔipaH0722*, or *ΔipaH0722* harboring *ipaH0722* or *ipaH0722CA*. The cell lysates prepared at the indicated time points were subjected to immunoblotting. Anti-actin antibody served as the loading control.

The infection of mice using the *ΔipaH*-chromosome mutant, which lacks all of the chromosomal *ipaH* genes, leads to an increased production of the NF-κB responsive gene MIP-2 [28]. In this current study, we used HeLa cells to determine the effect of the *ΔipaH*-chromosome mutant on the NF-κB activation by measuring the kinetics of IκBα degradation. HeLa cells were infected with YSH6000 (*S. flexneri* WT) or *ΔipaH*-chromosome mutant, and then whole cell lysates were harvested at 20, 40, 60, and 80 min post-infection for the detection of IκBα. The degradation rate of IκBα in cells infected with the *ΔipaH-chromosome* mutant was higher than that of WT, indicating that one or more of the chromosomal IpaH proteins contributed to the dampening of IκBα degradation (Fig. 1A, left). To identify the IpaH proteins that were involved in suppressing NF-κB activation, we generated deletions mutants for each of the seven chromosomal *ipaH* genes. IpaH0722 was critical for the inhibition of NF-κB activation. The degradation rate of IκBα was higher in *ΔipaH0722* infection compared to WT infection (Fig. 1A, right). IpaH0722, which corresponds to ORF SF0722 of *Shigella flexneri* 2a Sf301 strain, has a Cys residue in its C-terminal region that is required for E3 ubiquitin ligase activity [16], [28], [29].

In Cos-7 and HeLa cells, ectopically-expressed IpaH0722 localized to the cell membrane (Fig. 1B and S1A). A mouse lung infection model was used to evaluate the role of IpaH0722 in the pathogenesis of *Shigella*. The survival rates of mice infected with the *ΔipaH0722* mutant *Shigella* were increased compared to mice infected with WT *Shigella* (Fig. 1C). Next, to investigate whether the Cys379 residue in the IpaH0722 C-terminal region and its E3 ubiquitin ligase activity contributed to the pathogenesis of *Shigella*, we substituted Cys379 residue (IpaH0722CA). When 293T cells expressing IpaH0722 or IpaH0722CA were infected with *Shigella*, the activation of NF-κB was decreased in the presence of IpaH0722 but not IpaH0722CA (Fig. 1D). In addition, to confirm the importance of the E3 ubiquitin ligase activity of IpaH0722 in *Shigella* infection, HeLa cells were infected with *Shigella ΔipaH0722* harboring *ipaH0722* (Δ*ipaH0722/ipaH0722*) or *ipaH0722CA* (Δ*ipaH0722/ipaH0722CA*). In cells infected with the WT strain or Δ*ipaH0722/ipaH0722* strain, the degradation rate of IκBα was reduced at 60 and 80 min after infection compared to the Δ*ipaH0722* or *ΔipaH0722/ipaH0722CA* strains (Fig. 1E). It is important to note that IpaH0722 had no effect on Elk-1 and JNK activation (Fig. 1D and E). We speculated that IpaH0722 plays a role in the inhibition of *Shigella*-induced NF-κB activation in an E3 ubiquitin ligase-dependent manner.

### IpaH0722 selectively inhibits phorbol myristate acetate (PMA)-induced NF-κB activation

**Figure 2:**
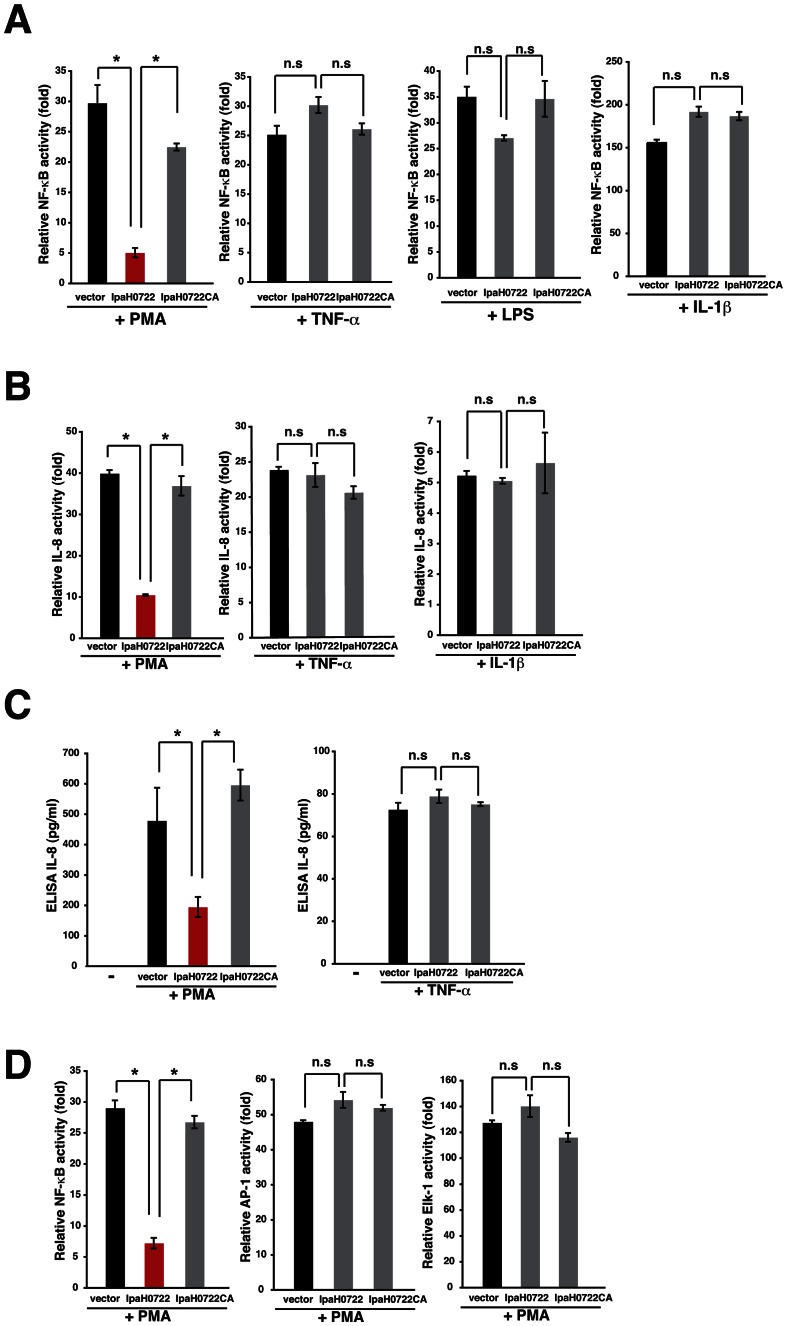
IpaH0722 selectively inhibits PMA–induced activation of the NF-κB pathway. (A) Luciferase reporter assays of 293T cells transiently transfected with an NF-κB reporter plasmid and empty vector, IpaH0722, or IpaH0722CA expressing plasmids. After 24 h, cells were treated with TNF-α, PMA, LPS, or IL-1β for 3 h and luciferase activity was measured. Results are presented as fold change relative to the activity of uninfected or unstimulated cells. *P<0.01 (B) IL-8 expression in 293T cells. Cells expressing empty vector, IpaH0722, or IpaH0722CA expressing plasmids were treated with TNF-α, PMA, or IL-1β for 3 h. Measurement of IL-8 reporter activity after PMA, TNF-α, and IL-1β stimulation. Results are presented as fold change relative to the activity of uninfected or unstimulated cells. *P<0.01. (C) The levels of IL-8 production were measured by ELISA. Cells expressing empty vector, IpaH0722, or IpaH0722CA expressing plasmids were treated with PMA and TNF-α, then supernatants were harvested for ELISA analysis. *P<0.0. (D)Luciferase reporter assays of 293T cells transiently transfected with NF-κB, AP-1, or Elk-1 reporter plasmids plus empty vector, IpaH0722, or IpaH0722CA expressing plasmids. After 24 h, cells were treated with PMA for 3 h and luciferase activity was measured. Results are presented as fold change relative to the activity of uninfected or unstimulated cells.*P<0.01, n.s., not significant.

NF-κB activity can be stimulated by multiple signaling pathways that are triggered by various receptors in response to exogenous and endogenous stimuli, including TNFα-TNFR, TLRs, and NLRs [30]. To characterize IpaH0722-dependent dampening of NF-κB activation, we measured the levels of NF-κB activation in IpaH0722- or IpaH0722CA-expressing 293T cells that were stimulated with PMA, TNF-α, lipopolysacharide (LPS), or IL-1βusing NF-κB-luciferase reporter assays. IpaH0722 markedly impaired PMA-dependent, but not TNF-α-, LPS-, and IL-1β-dependent NF-κB activation in an E3 ubiquitin ligase activity-dependent manner (Fig. 2A). To confirm the specificity of IpaH0722-mediated induction of NF-κB activation, we investigated the expression of IL-8 in 293T cells stimulated with PMA using an IL-8 reporter assay. Consistent with the NF-κB findings, IpaH0722 preferentially inhibited the PMA-dependent IL-8 expression (Fig. 2B). Moreover, the preferential targeting of PMA- but not TNF-α-induced NF-κB activation was also demonstrated by ELISA assays (Fig. 2C). Although PMA was also known to induce MAPK activation, IpaH0722 failed to inhibit PMA-dependent AP-1 and Elk-1 activation (Fig. 2D). Taken together these results clearly indicated that IpaH0722 preferentially inhibited PMA-induced NF-κB activation by targeting factors that modulated the NF-κB signaling pathway.

### Ectopically-expressed IpaH0722 localizes to the cell membrane

It has recently reported that *Salmonella* SspH2, an IpaH cognate protein, localizes to the host cell membrane through its modification by cellular palmitoyl transferases. SspH2 has a putative *S*-palmitoylation motif in its N-terminal and undergoes modification by host cell palmitoyltransferases, resulting in its localization to the host cell membrane [31]. Since we found a putative *S*-palmitoylated motif in IpaH0722 N-terminal portion, we investigated whether this motif played a role in IpaH0722 localization to the cell membrane. In Cos-7 and HeLa cells, ectopically-expressed IpaH0722 localized to host cell membrane (Fig. 1B and S1A). IpaH0722 has two Cys residues in its palmitoylation motif (Cys14 and Cys18), we generated constructs in which the Cys residues were substituted with Ser singly and in tandem (IpaH0722-C14S, -C18S, and -C14S/C18S). These constructs were ectopically expressed in HeLa cells to evaluate the subcellular localization of IpaH0722. IpaH0722 WT and IpaH0722CA localized to the cell membrane, whereas IpaH0722-C14S, -C18S, and -C14S/C18S accumulated within the cytosol. Therefore, palmitoylation at Cys14 and Cys18 residues of IpaH0722 are critical for its localization to the plasma membrane (Fig. S1A).

We next investigated whether membrane localization of IpaH0722 was required for its inhibition of NF-κB. The various IpaH0722 constructs were ectopically expressed in 293T cells, which were stimulated with PMA, NF-κB activity was assessed using NF-κB-luciferase reporter assays. IpaH0722, but not IpaH0722CA, inhibited NF-κB activation (Fig. S1B). Moreover, since the disruption of the putative palmitoylation sites in IpaH0722 mutants did not alter their ability to reduce PMA-dependent NF-κB activation, we presumed that the localization of IpaH0722 to the plasma membrane was not required for NF-κB inhibition in our experimental setting (Fig. S1B).

### Membrane rupture of epithelial cells by *Shigella* invasion triggers PKC–NF-κB activation

**Figure 3:**
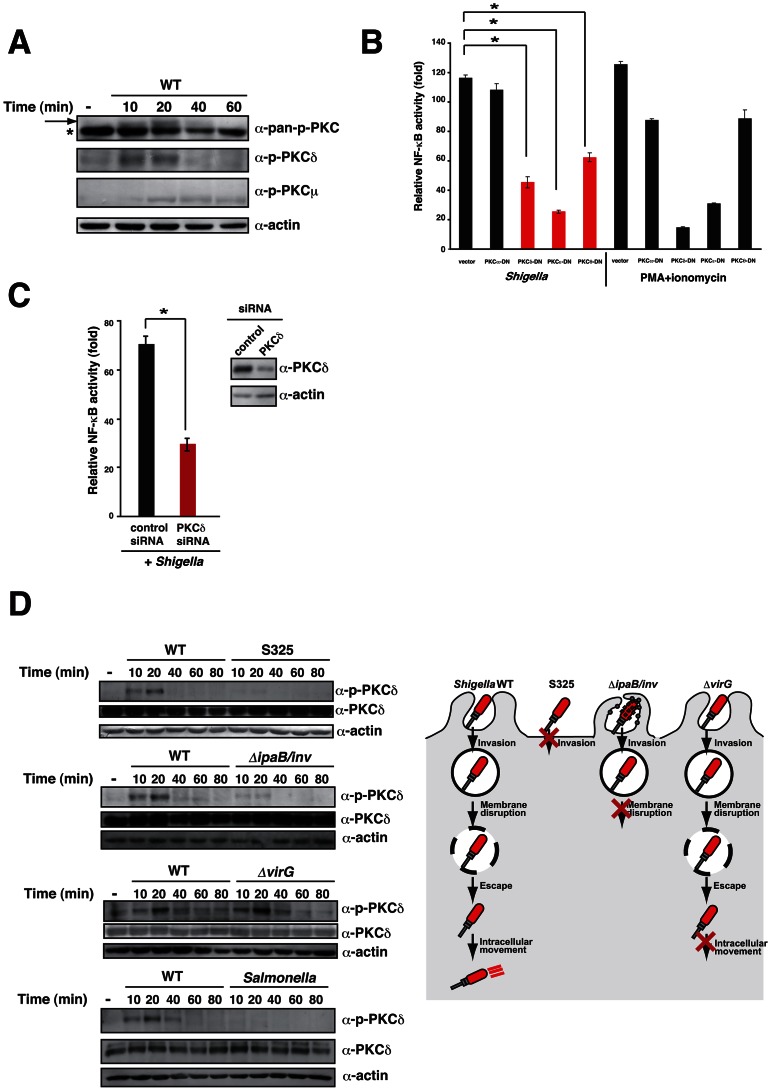
*Shigella* triggers PKC–NF-κB activation. (A) HeLa cells were infected with *Shigella*. Cell lysates were prepared at the indicated time points and subjected to immunoblotting with anti-phospho-PKC antibody. *non-specific bands. (B) NF-κB luciferase assays of 293T cells transiently transfected with an NF-κB reporter plasmid and empty vector or dominant-negative forms of PKC. After 24 h, cells were infected with *Shigella* or treated with PMA for 3 h, and luciferase activity was measured. Results are presented as fold change relative to the activity of uninfected or unstimulated cells. *P<0.01. (C) Cells were treated with PKCδ siRNA and transiently transfected with an NF-κB reporter plasmid. The cells were infected with *Shigella* and NF-κB reporter activity was measured. (D) *left*; HeLa cells were infected with *Shigella* WT, invasin expressing *ΔipaB* mutant, *ΔvirG* mutant, or *Salmonella*. The cell lysates were harvested at the indicated time points and subjected to immunoblot. *right*: A model of *Shigella* invasion into the epithelial cells.

PMA mimics the role of diacylglycerol (DAG) in the activation of the protein kinase C (PKC)-NF-κB pathway [32]. Therefore, we hypothesized that IpaH0722 selectively targeted the DAG–PKC–NF-κB pathway, which was likely due to the membrane localization of IpaH0722 once it was secreted by invading *Shigella* into epithelial cells. We sought to determine which of the PKC isoforms were involved in the activation of NF-κB. The PKC family of proteins is composed of 12 isoforms that act as lipid-activated Ser/Thr kinases [33]. The PKC family can be divided into four functional protein classes: conventional PKC (PKCα, β, and γ), novel PKC (PKCδ, ε, η, and θ), atypical PKC (PKCζ, λ/ι) and PKC related kinase (PKCμ). Conventional and novel PKCs have a DAG binding domain. Conventional PKCs require DAG and Ca^2+^ for their activation, whereas novel PKCs require only DAG for their activation. Atypical PKCs do not depend on DAG and Ca^2+^ for their activation. Since the activity of PKC is regulated by phosphorylation and its recruitment to the cell membrane, we investigated the levels of phosphorylated PKC during *Shigella* infection of HeLa cells. HeLa cells were infected with *Shigella* WT and cell lysates were harvested at 10, 20, 40, and 60 min after infection for the analysis of phosphorylated PKC by immunoblotting. As shown in Fig. 3A, *Shigella* infection augmented the phosphorylation of conventional or novel PKCs, such as PKCδ, at 10 and 20 min, and PKCμ at 20, 40, and 60 min post-infection.

To investigate whether PKC activation triggers NF-κB activation in *Shigella* infection, we exploited dominant negative forms (DN) of PKC and siRNA that targeted PKC. To investigate the role of PKC in the activation of NF-κB during *Shigella* infection, DN-PKCα and DN-PKCδ, ε, θ, were ectopically expressed in 293T cells. The DN-PKCδ, ε, θ, but not DN-PKCα, significantly decreased *Shigella*-induced NF-κB activation (Fig. 3B) suggesting that the PKCδ-NF-κB pathway plays a critical role during *Shigella* infection. Similarly, siRNA-mediated knockdown of PKCδ in 293T cells infected with *Shigella* decreased NF-κB activity to less than half of the control levels (Fig. 3C).

The invasion of epithelial by *Shigella* produces membrane ruffles through the remodeling of the F-actin cytoskeleton [34]–[36]. Immediately following bacterial invasion, the bacteria are rapidly surrounded by a vacuolar membrane that the bacteria disrupt to facilitate dissemination into the cytoplasm by inducing actin polymerization [34]–[36]. To further understand the mechanism of PKC activation by *Shigella* invasion, we examined PKC phosphorylation during *Shigella* infection. First we confirmed the importance of *Shigella* invasiveness. When HeLa cells were infected with *Shigella* WT or the T3SS deficient mutant S325, *Shigella* WT infection triggered the phosphorylation of PKCδ, however the S325 mutant did not (Fig. 3D). We next sought to determine whether vacuolar membrane disruption potentiated DAG–PKC–NF-κB signaling. We previously created an *ΔipaB/inv* mutant by introducing *Yersinia invasin* gene into the *ΔipaB* mutant [37]. The resulting *ΔipaB/inv* mutant was internalized into the endocytic vacuole and it was unable to disrupt the vacuolar membrane for dissemination into the cytoplasm (Fig. 3D). When HeLa cells were infected with *Shigella* WT or the *ΔipaB/inv* mutant, *Shigella* WT infection triggered the phosphorylation of PKCδ, however the *ΔipaB/inv* mutant did not (Fig. 3D). In contrast, both WT and *ΔvirG* mutant, which is unable to support intra- and inter-cellular movement, induced PKCδ phosphorylation (Fig. 3D). To further confirm the importance of vacuolar membrane disruption, we investigated PKC phosphorylation during *Salmonella* infection using a strain that triggers membrane ruffling but remain sequestered in the phagosome. When HeLa cells were infected with *Shigella* WT or the *Salmonella* WT, *Shigella* WT but not *Salmonella* WT infection triggered the phosphorylation of PKCδ (Fig. 3D). These data supported the notion that membrane rupture by *Shigella* in infected epithelial cells triggers the activation of the DAG-PKC-NF-κB pathway.

### IpaH0722 inhibits the PKC–NF-κB pathway without targeting the CBM complex

**Figure 4:**
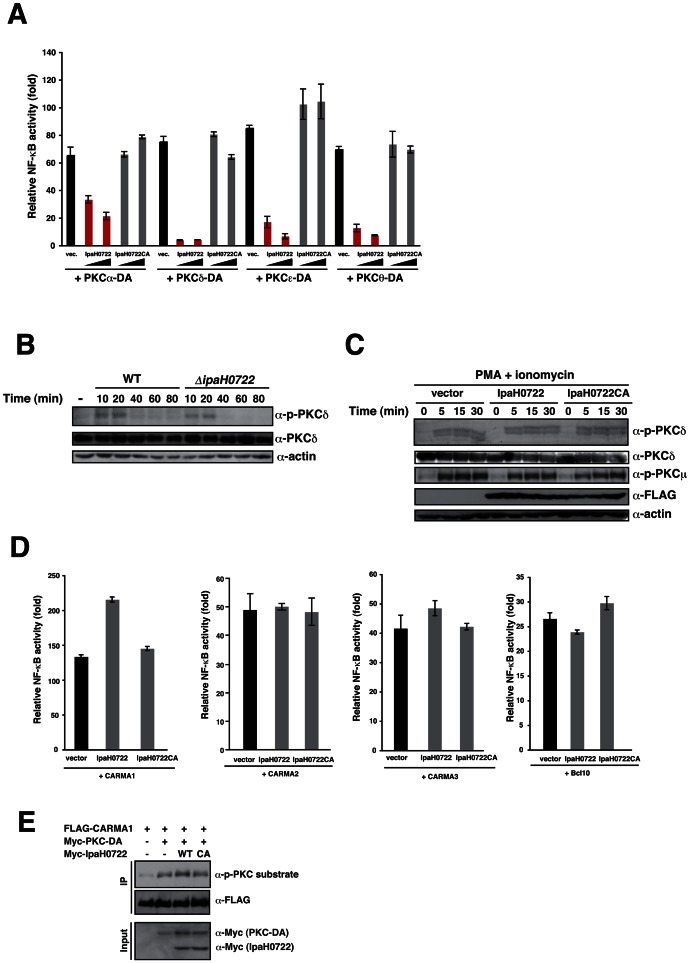
IpaH0722 targets PKC–NF-κB activation but not via the CBM complex. (A) IpaH0722 inhibits PKC-induced NF-κB activation. Luciferase assays of 293T cells that were transiently transfected with dominant-active forms of PKC and empty vector, IpaH0722, or IpaH0722CA expressing plasmids. Results are presented as fold change relative to the activity of uninfected or unstimulated cells. *P<0.01. (B) HeLa cells were infected with *Shigella* WT or the *ΔipaH0722* mutant. The cell lysates were harvested at the indicated time points and subjected to immunoblotting. (C) Cells that transiently expressed empty vector, IpaH0722, or IpaH0722CA were treated with PMA+ionomycin for the indicated time points. Cell lysates were subjected to immunoblotting. (D) Luciferase reporter assays of 293T cells were transiently transfected with NF-κB and empty vector, IpaH0722, or IpaH0722CA expressing plasmids. Results are presented as fold change relative to the activity of uninfected or unstimulated cells. (E) 293T cells were transfected with FLAG-CARMA1 with or without PKCδ-DA, Myc-IpaH0722, or Myc-IpaH0722CA. After 24 h, cells were harvested and subjected to immunoprecipitation (IP).

Because *Shigella* invasion of epithelial cells triggers the DAG–PKC–NF-κB signaling, we sought to determine the host factors in the PKC–NF-κB signaling pathway that were targeted by IpaH0722 E3 ubiquitin ligase. First, we determined which of the steps during the activation of PKC–NF-κB was targeted by IpaH0722. The NF-κB activity induced by PKC signaling factors was measured in 293T cells with ectopic expression of IpaH0722 or IpaH0722CA. The transiently transfected cells were stimulated with a subset of constitutively active PKC isoforms (PKCα, -δ, -ε, and θ) and NF-κB activity was determined. IpaH0722, but not IpaH0722CA, inhibited all PKC isoforms (PKCα, -δ, -ε, and -θ) and NF-κB activation, indicating that IpaH0722 targeted PKC itself or factors that lie directly downstream of PKC (Fig. 4A). In HeLa cells, similar levels of phosphorylated PKCδ were detected in response to *Shigella* WT and *ΔipaH0722* infection (Fig. 4B). Moreover, no differences were detected in PKC phosphorylation in PMA plus ionomycin stimulated 293T cells expressing IpaH0722 or IpaH0722CA (Fig. 4C). In addition, IpaH0722 did not bind to PKC, and had no effect on protein stability of PKC, suggesting that IpaH0722 targets downstream of PKC rather than PKC itself (Fig. S2). We therefore focused on the CARMA-Bcl10-MALT1 (CBM) complex because the phosphorylation of CARMA1, 2, and 3 by PKC induces a conformational change that recruits Bcl10-MALT1 to CARMA (CBM signalosome), which is essential for the activation of downstream signaling [38], [39]. Therefore, we tested the effect of IpaH0722 on CBM complex formation, and found that IpaH0722 failed to inhibit CARMA1-, CARMA2-, CARMA3-, or Bcl10-induced NF-κB activity (Fig. 4D). Indeed, IpaH0722 did not bind to CARMA1-, CARMA2-, CARMA3-, or Bcl10, and had no effect on their protein stability (Fig. S3). Furthermore, since IpaH0722 did not affect PKC-mediated CARMA phosphorylation (Fig. 4E), we believe that IpaH0722 inhibited the PKC–NF-κB pathway but not via the CBM complex.

### IpaH0722 preferentially inhibits TRAF2-mediated NF-κB activation

**Figure 5:**
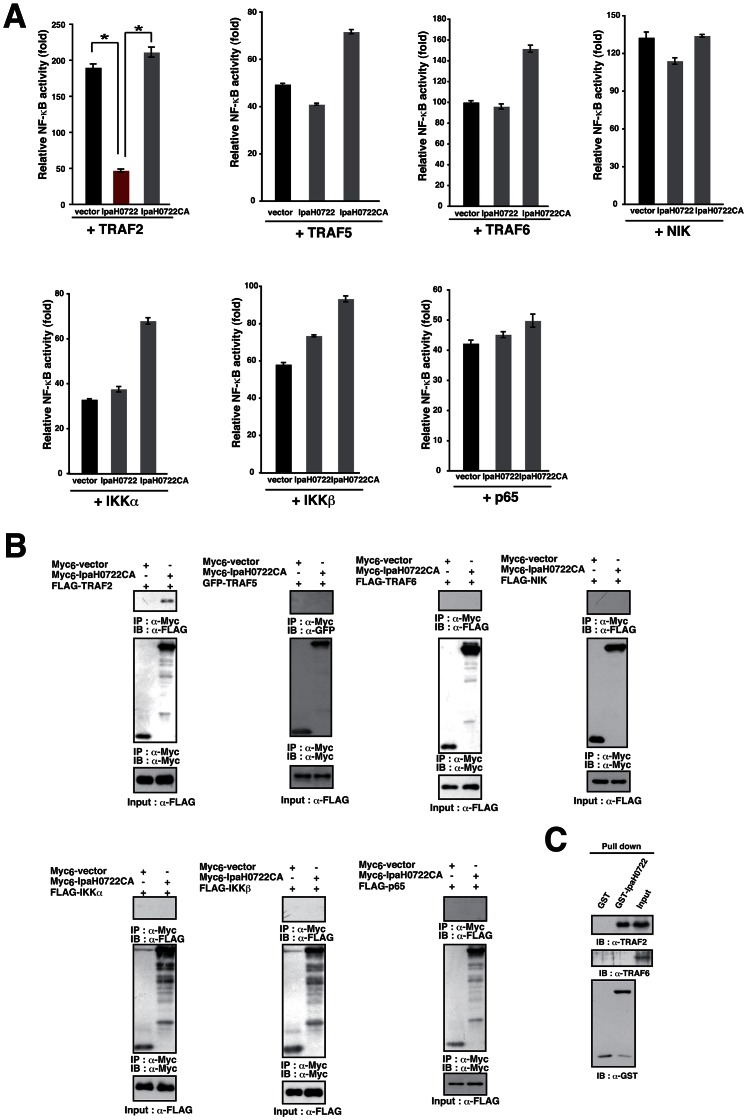
IpaH0722 inhibits TRAF2-mediated NF-κB activation. (A) Luciferase reporter assays of 293T cells transiently transfected with NF-κB signaling factors and empty vector, IpaH0722, or IpaH0722CA expressing plasmids. Results are presented as fold change relative to the activity of uninfected or unstimulated cells. *P<0.01. (B) 293T cells were transfected with FLAG or GFP-tagged NF-κB signaling factor with or without Myc_6_-IpaH0722CA. After 24 h, cells were harvested and subjected to immunoprecipitation (IP) and immunoblotting. (C) Pull down assays using GST or GST-IpaH0722 were performed with HeLa whole cell lysates. Samples were subjected to immunoblotting with anti-TRAF2 and -TRAF6.

We subsequently investigated other NF-κB signaling factors, namely TRAF2, TRAF5, TRAF6, NIK, IKKα, IKKβ, or p65 as potential IpaH0722 targets [40]. To this end, 293T cells expressing NF-κB-luciferase reporter gene together with TRAF2, TRAF5, TRAF6, NIK, IKKα, IKKβ, or p65 were transfected with a vector encoding IpaH0722 or IpaH0722CA and luciferase activity was measured (Fig. 5A). IpaH0722, but not IpaH0722CA, inhibited NF-κB activity when the cells that expressed TRAF2 suggesting that TRAF2 is a target for IpaH0722 E3 ubiquitin ligase (Fig. 5A). In previous reports, PKC-mediated phosphorylation of TRAF2 was a prerequisite for NF-κB activation in epithelial cells [41], [42]. PKCδ and PKCε phosphorylated TRAF2 at residue Thr117 resulting in IKK recruitment and ultimately NF-κB activation [41], [42]. We therefore used siRNA-mediated TRAF2 knockdown cells to measure the effect of TRAF2 on activation of the PKC-NF-κB pathway. The results showed that TRAF2 knockdown decreased NF-κB activation in response to PMA stimulation and *Shigella* infection (Fig. S4A). To further confirm the effect of TRAF2 knockdown on NF-κB activation we utilized *Traf2*-knockout mouse embryonic fibroblasts (MEF) [43]. We transduced *Traf2* into the *Traf2*-knockout MEFs by retrovirus infection to generate stably expressing *Traf2* cell lines. The degradation of IκBα in *Traf2* stably expressing MEF cells and *Traf2*-knockout MEF cells during *Shigella* infection was determined. As shown in Fig. S4B, the degradation rate of IκBα in *Traf2* stably expressing MEFs was higher than that of *Traf2*-knockout MEFs at 20 and 40 min after infection suggesting that TRAF2 plays a role in *Shigella*-induced NF-κB activation.

Having shown that IpaH0722 targets TRAF2, we performed immunoprecipitation assays to determine whether IpaH0722 interacted with TRAF2. Cell lysates were harvested from 293T cells that expressed IpaH0722CA and TRAF2, TRAF5, TRAF6, NIK, IKKα, IKKβ, or p65. IpaH0722 precipitated with TRAF2, but not TRAF5, TRAF6, NIK, IKKα, IKKβ, or p65 (Fig. 5B). The interaction between IpaH0722 and TRAF2 was further confirmed by GST-pull down assay. Using whole cell lysates of HeLa and GST-IpaH0722, we were able to pull down endogenous TRAF2, but not TRAF6 (Fig. 5C).

In previous reports, structural analysis of IpaH proteins using IpaH1.4 or IpaH3 revealed that the LRR region of the IpaH family is required for substrate recognition, while the CTR region is essential for its E3 ubiquitin ligase activity [44], [45]. IpaH proteins share characteristic domains: an N-terminal 60–70 amino acid stretch, a LRR region, and an intervening region flanked by the LRR and the conserved CTR (Fig. S5A). We constructed a series of GST-tagged IpaH0722 truncations and performed GST-pull down assays using HeLa whole cell lysates. IpaH0722 truncations that contained the LRR regions bound to TRAF2; however, IpaH0722 truncations that contained the N-terminal, intervening region, or CTR failed to bind to TRAF2 (Fig. S5B).

### IpaH0722 targets TRAF2 for ubiquitination and proteasomal degradation

**Figure 6:**
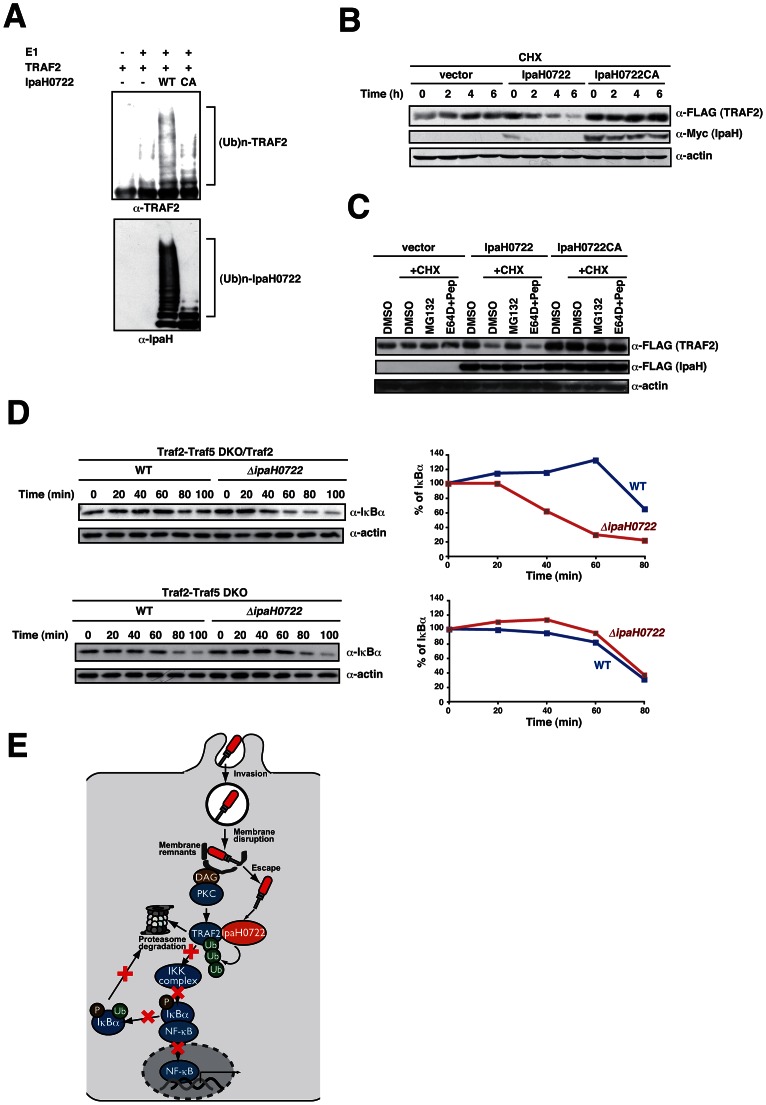
IpaH0722 targets TRAF2 for ubiquitination. (A) *In vitro* ubiquitination assay using TRAF2 and a mixture of E1, UbcH5b, ATP, and ubiquitin in the presence or absence of IpaH0722 or IpaH0722CA. (B) 293T cells were transfected with FLAG-TRAF2 with and without Myc-IpaH0722 or Myc-IpaH0722CA. After 24 h, the cells were treated with CHX (50 mg/ml) and cell lysates were prepared at the indicated time points. (C) 293T cells were transfected with FLAG-TRAF2 with and without FLAG-IpaH0722 or FLAG-IpaH0722CA. After 24 h, the cells were treated with DMSO or CHX (50 mg/ml) plus MG132 or E64D+pepstatin A. Cell lysates were prepared after 3 h and samples were subjected to immunoblotting. (D) *Traf2/Traf5* ^−/−^ MEFs and *Traf2/Traf5* ^−/−^ MEFs stably expressing *Traf2* were infected with *Shigella* WT or *ΔipaH0722*. Cell lysates were harvested at the indicated time point and subjected to immunoblotting with anti- IκBα Anti-actin antibody was used as a loading control. (E) A model of this study. *Shigella* invasion induced phagosome membrane rupture of epithelial cells, which stimulates DAG–PKC–NF-κB activation. However, *Shigella* deliver IpaH0722 to inhibit PKC–NF-κB activation by ubiquitinating TRAF2 and promoting its proteasome degradation.

Since IpaH0722 inhibited NF-κB activation in an E3 ubiquitin ligase-dependent manner, we tested whether IpaH0722 ubiquitinated TRAF2 using an *in vitro* ubiquitination assay. TRAF2, IpaH0722, or IpaH0722CA (purified from *E. coli*) were combined with E1, ATP, UbcH5b (an E2 ubiqitin conjugating enzyme) in assay medium and subjected to immunoblotting. TRAF2 was ubiquitinated by IpaH0722 but not IpaH0722CA (Fig. 6A). To confirm the fate of ubiquitinated TRAF2, we measured the stability of TRAF2 in 293T cells ectopically expressing IpaH0722 or IpaH0722CA and treated with cycloheximide (CHX; a protein synthesis inhibitor) for 2, 4, and 6 h. TRAF2 degradation was faster in IpaH0722 expressing cells compared to IpaH0722CA expressing cells, which confirmed that IpaH0722 targeted TRAF2 for ubiquitin-mediated protein degradation (Fig. 6B). We also measured TRAF2 degradation in 293T cells that were co-transfected with TRAF2 plus IpaH0722 or IpaH0722CA, and treated with MG132 (a proteasome inhibitor) or E64D plus pepstain A (lysosome inhibitors). As shown in Fig. 6C, MG132 treatment prevented TRAF2 degradation in the presence of IpaH0722, whereas E64D plus pepstatin A treatment did not prevent TRAF2 degradation. These data confirm that IpaH0722 targets TRAF2 for ubiquitination and proteasomal degradation.

Previous studies indicated that TRAF5 compensates for TRAF2 function [43], so we exploited *Traf2/Traf5* double knockout MEFs to clarify the requirement of TRAF2 on NF-κB activation. We introduced *Traf2* into the *Traf2/Traf5* double knockout MEFs by retrovirus transduction. Under puromycin selection, we obtained stable TRAF2-expressing *Traf2/Traf5* double knockout MEFs. *Traf2/Traf5* double knockout MEFs and stable *Traf2*-expressing *Traf2/Traf5* double knockout MEFs were infected with *Shigella* WT or *ΔipaH0722*, and IκBα degradation was measured over time. The degradation rate of IκBα in *ΔipaH0722*-infected stable TRAF2-expressing *Traf2/Traf5* double knockout MEFs was higher compared to WT *Shigella* infection (Fig. 6D). In contrast, the degradation rates of IκBα in WT and *ΔipaH0722*-infected double knockout MEFs were almost equal (Fig. 6D). To evaluate the importance of the *in vivo* role of E3 ligase activity of IpaH0722 for *Shigella* virulence, we infected mice with a sublethal dose of *Shigella* WT, *ΔipaH0722, ΔipaH0722/0722*, or *ΔipaH0722/0722CA*. Mice infected with *ΔipaH0722 or ΔipaH0722/0722CA* mutants showed significantly reduced bacterial colonization when compared to that of WT or *ΔipaH0722/0722* infection (Fig. S6A). Furthermore, histopathology showed that inflammatory responses, such as suppurative masses, neutrophil infiltration, and macrophage infiltration, were significantly higher in mice infected with *ΔipaH0722* or *ΔipaH0722/0722CA* mutants when compared to that of WT or *ΔipaH0722/0722* infections (Fig. S6B). These results supported our notion that the interaction between IpaH0722 and TRAF2 is an important mechanism for dampening NF-κB activation during *Shigella* infection of epithelial cells.

## Discussion

In this study we show that IpaH0722, a chromosomally-encoded IpaH E3 ligase, specifically dampens the PKC-dependent activation of NF-κB in response to *Shigella* invasion of epithelial cells. We find the IpaH0722 exerts its effect by targeting TRAF2 for proteasomal-dependent degradation (Fig. 6E). The inhibitory activity possessed by IpaH0722 appears to be an important mechanism for the downregulation of the acute inflammatory response by blocking the activation of PKC-NF-κB pathway. This is a critical step since cell membrane rupture is a part of the process of *Shigella* invasion into epithelial cells, and the recognition of this as a DAMP triggers the recruitment and activation PKC, and ultimately the activation of the innate immune response via PKC-NF-κB pathway. This notion was supported by the experiments using the *ΔipaB/inv* mutant, which was internalized by the vacuolar membrane but could not rupture the vacuole [36], we showed that *Shigella* WT infection, but not *ΔipaB/inv* mutant infection, caused in the phosphorylation of PKCδ (Fig. 3E). In fact, Dupont et al. recently reported that the vacuolar membrane remnants generated by *Shigella* invasion into epithelial cells undergo ubiquitination and provided a cue to stimulate autophagy and inflammatory responses [46]. Moreover, Shahnazari et al. showed that the DAG generated in *Salmonella* containing vacuole (SCV) membranes serves as a specific signal to induce autophagy through recruitment of PKC into the SCV [47]. Tattoli et al. showed that vacuolar membrane damage triggers amino acid starvation and the downregulation of mTOR activity, thereby inducing autophagy against intracellular *Shigella* and *Salmonella* [48]. Phagosomal membrane disruption by *Listeria monocytogenes* LLO in macrophages, which is known as a cholesterol-dependent cytolysin, triggers the recruitment of PKC to the membrane and its activation [49]. After disruption of the *Listeria* phagosome the membrane damage was recognized by PKC. These data suggest that PKC functions as a part of the repair mechanism of host cells, and PKC recruitment by the membrane rupture is recognized as a DAMP by the innate immune system [49]. These studies also imply that the membrane rupture, which is created by bacterial infection, acts as a double-edged sword for the pathogens; on one hand it benefits the bacteria by promoting further dissemination and multiplication, and on the other it hinders the bacteria by acting as a DAMP that stimulates the host innate immune response. In line with this, we identified IpaH0722 as a *Shigella* effector that plays an important pathogenic role because it counteracts DAMPs-mediated innate immune responses.

Recently we reported that another *Shigella* effector, OspI targets UBC13 and dampens the DAG–CBM–TRAF6–NF-κB pathway, which is triggered by membrane ruffles around the *Shigella* entry sites into epithelial cells [12]. OspI acts as a glutamine deamidase for UBC13 to convert Q100 to E100, thus inactivating UBC13 E2 ubiquitin conjugating enzymatic activity that is required for TRAF6 activation [12]. Currently, although the reason underlying why *Shigella* deliver IpH0722 in addition to OspI during the invasion of epithelial cells remains partly unclear, we believe that it is due to the fact that the two different signaling pathways are required to neutralize the acute inflammatory response and are triggered by distinct bacteria-induced cellular events: DAG-CBM-TRAF6-NF-κB (membrane ruffles) and DAG-PKC-TRAF2-NF-κB (membrane rupture) [12; this study].

TRAF2 and TRAF5 have been extensively studied in TNFR–NF-κB signaling in immune cells, and it has also been reported that TRAF2 is essential in Nod1–, PKC–, and non-canonical NF-κB signaling in non-myeloid cells [42], [50], [51]. In previous studies, PKC-mediated phosphorylation of TRAF2 at Ser11, Ser55, and Thr117 was required for prolonged NF-κB activation, but not JNK activation [42], [52]–[54]. Consistent with these observations, our data showed that treatment of siRNA-mediated knockdown of *Traf2* decreased PMA-induced NF-κB activation, suggesting that TRAF2 is involved in PKC–NF-κB signaling (Fig. S4). According to Hasegawa et al., *Traf2/Traf5* double knockout MEFs but not *TRAF6* knockout MEFs had decreased NF-κB activation upon Nod1-RIP2 stimulation, suggesting that TRAF2 was also important in the Nod1–NF-κB pathway [51]. Since Nod1 recognizes the peptidoglycan that is released from *Shigella* at the entry site and in the cytoplasm, and plays an important role in *Shigella*-induced NF-κB activation, IpaH0722 likely inhibits both the PKC–NF-κB pathway and the Nod1–RIP2–TRAF2–NF-κB pathway during *Shigella* infection [55]–[57]. In fact, we see that IpaH0722 also inhibited Nod1-stimulated NF-κB activation in our reporter assay (data not shown). We have provided evidence that IpaH0722 interacted with TRAF2 via its substrate recognition site, LRR, and modified its ubiquitination (Fig. 6 and Fig. S5). The ubiquitination of TRAF2 by IpaH0722 led to its proteasomal-dependent degradation and inhibited NF-κB activation (Fig. 6). Though not yet fully understood, IpaH0722 does not inhibit NF-κB in response to TNF-α; unidentified conformational changes or signaling pathways involving TRAF2 likely occur during *Shigella* infection or PMA stimulation.

Herein, we provide the first evidence that *Shigella* invasion induced membrane rupture of epithelial cells, which stimulates PKC-NF-κB signaling. Moreover, *Shigella* used the E3 ubiqitin ligase effector, IpaH0722, to target TRAF2 for proteasomal-dependent degradation (Fig. 6E). This is the first report IpaH0722 as a non-plasmid-encoded T3SS effector protein in *Shigella*.

## Materials and Methods

### Strain and plasmids

*Shigella flexneri* strain YSH6000 was used as the wild type and S325 (*mxiA*::Tn*5*) was used as a T3SS-deficient negative control. *Salmonella typhimurium* SB300 strain was used as the wild-type strain [58]. Construction of the non-polar mutant of *ipaH-chromosome*, *ipaH0722, ipaB*, and *virG* of *S. flexneri* YSH6000 were carried out as described previously [28], [37]. The *ipaH0722-FLAG* or *ipaH0722CA-FLAG* were cloned into pWKS130 to yield p-*ipaH0722-FLAG* and p-*ipaH0722CA-FLAG*. The resultant plasmids were introduced into the *ΔipaH0722* strain. The *ipaH0722* or *ipaH0722CA* coding sequences were amplified by PCR and cloned into pCMV-FLAG, pEGFP, pGEX-4T-1, pcDNA-Myc_6_ (6× Myc), and pcDL-SRα-Myc vectors. cDNAs for human *Traf2*, *Traf5*, *Traf6*, *Carma1*, *Carma2*, *Carma3*, *Bcl10*, *IKKα*, *IKKβ*, *p65*, and a series of *Pkc* mutants were cloned into pCMV-FLAG, pEGFP, pcDNA-Myc_6_, pcDL-SRα-Myc, and pcDNA-FLAG vectors. cDNA for mouse *Traf2* was cloned into pGEX 4T-1. Site direct mutagenesis of *ipaH0722* or *Pkc* was performed using the QuickChange site directed mutagenesis kit (Stratagene).

### Materials

The anti-M2 FLAG monoclonal FLAG antibody (Sigma-Aldrich), anti-Myc 9B11, phospho-PKC (pan) (βII Ser660), phospho-PKD/PKCμ (Ser744/748), phospho-(Ser) PKC substrate, phospho-JNK antibody (Cell signaling), anti-actin (MILLIPORE), anti-PKCδ (C-20), PKCμ (C-20), TRAF2, TRAF6 antibody (Santa Cruz Biotechnology), and anti-IκBα (BD transduction) were obtained commercially. The anti-IpaH antibody was described previously [29]. PMA, LPS, ionomycin, E64D, and pepstatin A were obtained from Sigma. IL-1β and TNF-α were obtained from Peprotech. MG132 was obtained from Peptide Inst.

### Cell culture

HeLa cells were cultured in Eagle's minimal essential medium (Sigma) supplemented with 10% fetal calf serum. Cos-7 and 293T cells were cultured in Dulbecco's modified Eagle medium (Sigma) supplemented with 10% fetal calf serum. The *Traf2*-deficient or *Traf2/Traf5*-deficient MEFs were maintained as described previously [43]. To construct *Traf2* ^−/−^ or *Traf2/Traf5* ^−/−^cells stably expressing Traf2, cDNAs encoding these genes were subcloned into pMX-puro retroviral expression vectors. Retroviral supernatants were produced in Plat-E cells. Target cells were transduced with supernatants in the presence of DO-TAP (Roche) and then cloned under puromycin selection.

### Bacterial infection

HeLa cells were infected with various strains of *Shigella* at a multiplicity of infection (moi) of 100. In the case of the *Shigella* strains expressing afimbrial adhesin (Afa), cells were infected at moi of 10. To adjust the intracellular bacterial number, *ΔipaB/inv* were set to 3 times moi of WT. Infection was initiated by centrifuging the plate at 700× *g* for 10 min. After incubation for 20 min at 37°C, the plates were washed three times with PBS, transferred into fresh medium containing gentamicin (100 µg/ml) and kanamycin (60 µg/ml) to kill extracellular bacteria. *Salmonella* infection was conducted as described previously [58]. After incubation for indicated time, the cells were washed with PBS and harvested into 2× Laemmli's sample buffer for immunoblotting. The density of each band was quantified by measuring the mean intensity using NIH image software version 1.63 and the expression levels were normalized to the levels of β-actin. All immunoblotting experiments were repeated multiple times and representative data of similar results are presented.

### Luciferase reporter assays

293T cells were seeded into 24-well plates. After 24 h, the cells were transfected with reporter plasmids (pNF-κB-luc, pIL-8-luc, pElK-1-luc, or pAP-1-luc), Renilla luciferase constructs (phRL-TK, Promega) using FuGENE6 transfection reagent (Roche). The IL-8 reporter plasmid was constructed by amplifying the 181 bp human IL-8 promoter sequence from the genomic DNA of HeLa cells, which was then inserted into the pMet-luc reporter plasmid (Clontech). Equal amounts of empty vector were used to control for the transfection process. After 24 h, cells were infected with *Shigella* WT (moi = 30) or treated with PMA (25 nM), TNF-α (10 ng/ml) or LPS (100 ng/ml) for 3 h. To investigate LPS stimulation, TLR4 and MD2 vectors were also transfected into the cells. Cell extracts were prepared and reporter activity was determined using the luciferase assay system (Promega). Results are presented as fold-change relative to the activity of uninfected or unstimulated cells. Data are representative of three independent experiments.

### Expression and purification of recombinant proteins

For GST fusion proteins, *E. coli* BL21 (DE3) strain harboring pGEX4T-1 derivatives were cultivated in L-broth supplemented with ampicillin (50 µg/ml) 3 h at 30°C. Expression was induced by the addition of 1 mM IPTG and incubation for 3 h at 30°C. Bacteria were disrupted by sonication and lysozyme treatment. Purification of the GST fusion proteins with glutathione-Sepharose 4B (GE Healthcare) was performed according to the manufacturer's protocol.

### Pull-down assay

GST-IpaH0722 bound to glutathione sepharose 4B beads was mixed with HeLa cell lysates for 2 h at 4°C. After centrifugation, the beads were washed five times with 0.5% Triton X-100-PBS and subjected to immunoblotting. The immunoblotting experiments were repeated multiple times and representative data of similar results are presented.

### Immunoprecipitation

293T cells were transiently transfected using FuGENE 6 (Roche). Cells were washed with PBS and lysed for 30 min at 4°C in lysis buffer containing 150 mM NaCl, 50 mM HEPES pH 7.5, 1 mM EDTA, 0.5% NP-40, and Complete protease inhibitor cocktail (Roche). Lysates were cleared by centrifugation and proteins were immunoprecipitated 2 h with anti-Myc (9B11) or anti-M2 FLAG and Protein G beads (Sigma) at 4°C. Immunoprecipitates were washed five times with lysis buffer and subjected to immunoblotting. The immunoblotting experiments were repeated multiple times and representative data of similar results are presented.

### *In vitro* ubiquitination assays

*In vitro* TRAF2 ubiquitination assays were performed in 40 µl reaction mixture containing reaction buffer (25 mM Tris-HCl [pH 7.5], 50 mM NaCl, 5 mM ATP, 10 mM MgCl_2_, and 0.1 mM DTT), 1 µg TRAF2, 0.5 µg E1, 2 µg UbcH5b, and 2 µg ubiquitin purified from *E. coli* in the presence and absence of GST-IpaH0722 or GST-IpaH0722CA. Reactions were incubated at 37°C for 1 h and stopped by the addition of 5× Laemmli sample buffer. All immunoblotting experiments were repeated multiple times and representative data of similar results are presented.

### Cycloheximide chase assay

At 24 h after transfection, 293T cells were treated with cycloheximide (50 µg/ml) (Wako) for the indicated times and cell lysates were harvested for immunoblotting. The density of each band was quantified by measuring the mean intensity using NIH image software version 1.63. The expression levels were normalized to the levels of β-actin. All immunoblotting experiments were repeated multiple times and representative data of similar results are presented.

### RNAi

Human PKCδ- or human TRAF2-specific siRNA were prepared by Sigma as follows: 5′-CGUGUGGACACGCCACAUUAU-3′ and 5′-AAUGUGGCGUGUCCACACGGA-3′ (PKCδ) or 5′-GGCCAGUCAACGACAUGAACA-3′ and 5′-UUCAUGUCGUUGACUGGCCUC-3′ (TRAF2). Cells were transfected using RNAiMax (Invitrogen). siRNA treated cells were utilized after 72 h for further analyses.

### Murine pneumonia model

The pulmonary infection model in the mice was described previously [28]. In brief, five-week-old female Balb/c mice (CREA Japan) were housed in the animal facility of the Institute of Medical Science, University of Tokyo, in accordance with University guidelines. *S. flexneri* were suspended in sterile saline, and 20 µl of the bacterial suspension was administered intranasally at 1×10^8^ cfu (survival assay) or 1×10^7^ cfu (colonized bacterial number assay). For histological analysis, mice were sacrificed at 24 h after infection, and their lungs were harvested, fixed in 4% paraformaldehyde in PBS, and frozen in liquid nitrogen for sectioning. The sections were stained with hematoxylin and eosin and examined under a microscope. Viable bacteria in the lung tissue were counted by culturing homogenized tissue for 18 h on LB agar plates. Each data point is the mean of the values for 6 infected mice in each group.

### Ethics statement

All animal experiments were carried out in strict accordance with the University of Tokyo's regulations for Animal Care and Use protocol, which was approved by the Animal Experiment Committee of the Institute of Medical Science, the University of Tokyo (approval number; PA11-92). The committee acknowledged and accepted both the legal and ethical responsibility for the animals, as specified in the Fundamental Guidelines for Proper Conduct of Animal Experiment and Related Activities in Academic Research Institutions under the jurisdiction of the Ministry of Education, Culture, Sports, Science and Technology, 2006 (Japan). All surgery was performed under carbon dioxide euthanasia, and all efforts were made to minimize suffering.

### Statistical analysis

Values are reported as means ± standard deviation (SD) or means ± standard error (SEM) of data obtained for independent experiments. Statistical analysis was performed Student's *t*-test or a one-way ANOVA. Kaplan-Meier survivor curves were generated. p-values<0.05 were considered significant.

## Supporting Information

Figure S1 **IpaH0722 localizes to host cell membrane.** (A) A series of GFP-tagged IpaH0722 mutants were transiently expressed in HeLa cells and their cellular localization was examined by immunofluorescence analysis. (B) Luciferase reporter assays of 293T cells transiently transfected with an NF-κB reporter plasmid and an empty vector or a series of IpaH0722 mutants. After 24 h, the cells were treated with PMA for 3 h and luciferase activity was measured. Results are presented as fold change relative to the activity of uninfected or unstimulated cells. *P<0.01.
(EPS)

Figure S2 **IpaH0722 targets PKC–NF-κB activation but not PKC itself.** (A) 293T cells were transfected with series of Myc_6_-tagged PKC with FLAG-IpaH0722CA. After 24 h, cells were harvested and subjected to immunoprecipitation (IP) and immunoblotting. (B) 293T cells were transfected with series of PKC with and without IpaH0722 or IpaH0722CA. After 24 h, the cells were treated with CHX (50 mg/ml) and cell lysates were prepared at the indicated time points. In the case of PKCδ, endogenous PKCδ levels were detected.
(EPS)

Figure S3 **IpaH0722 did not target the CBM complex.** (A) 293T cells were transfected with FLAG-tagged CARMA1-, CARMA2-, CARMA3-, or Bcl10 with or without Myc_6_-IpaH0722CA. After 24 h, cells were harvested and subjected to immunoprecipitation (IP) and immunoblotting. (B) 293T cells were transfected with FLAG-tagged CARMA1-, CARMA2-, CARMA3-, or Bcl10 with and without FLAG-IpaH0722 or FLAG-IpaH0722CA. After 24 h, the cells were treated with CHX (50 mg/ml) and cell lysates were prepared at the indicated time points.
(EPS)

Figure S4 **TRAF2 is involved in PMA and** ***Shigella*** **-induced NF-κB activation.** (A) Cells with siRNA-mediated knockdown of TRAF2 were transiently transfected with an NF-κB reporter plasmid. These cells were stimulated PMA or infected with *Shigella* for 3 h and NF-κB reporter activity was measured. (B) *Traf2* ^−/−^ MEFs and *Traf2* ^−/−^ MEFs stably expressing *Traf2* were infected with *Shigella*. Cell lysates were harvested at the indicated time points and subjected to immunoblotting with anti- IκBα. Anti-actin antibody was used as a loading control.
(EPS)

Figure S5 **The LRR region of IpaH0722 is required for TRAF2 binding.** (A) Schematic representation of full length and truncated IpaH0722. (B) GST-tagged IpaH0722 or IpaH0722 truncated forms were prepared from *E. coli* and used to GST pull down assays with HeLa whole cell lysates.
(EPS)

Figure S6 **IpaH0722 modulates the host inflammatory response in a murine model of pneumonia infection.** (A)The number of viable bacteria in the lungs of mice infected with the *S. flexneri* WT strain, *ΔipaH0722* mutant, *ΔipaH0722/ipaH0722* complementation strain, or *ΔipaH0722/ipaH0722CA* complementation strain at 24 h after inoculation. Data are the mean values of 6 mice per group (±SD). *P<0.05 (B) Histological analysis of mouse lungs at 24 h after inoculation. Sections of infected lungs were stained with hematoxylin and eosin.
(EPS)

## References

[1] Chen GY, Nuñez G (2010) Sterile inflammation: sensing and reacting to damage. Nat Rev Immunol 10: 826–837. 21088683 10.1038/nri2873 PMC3114424

[2] Davis BK, Wen H, Ting JP (2011) The inflammasome NLRs in immunity, inflammation, and associated diseases. Annu Rev Immunol 29: 707–735. 21219188 10.1146/annurev-immunol-031210-101405 PMC4067317

[3] Ashida H, Ogawa M, Kim M, Mimuro H, Sasakawa C (2012) Bacteria and host interactions in the gut epithelial barrier. Nat Chem Biol 8: 36–45. 10.1038/nchembio.741 22173358

[4] Galán JE (2009) Common themes in the design and function of bacterial effectors. Cell Host Microbe 5: 571–579. 19527884 10.1016/j.chom.2009.04.008 PMC2729653

[5] Rahman MM, McFadden G (2011) Modulation of NF-κB signalling by microbial pathogens. Nat Rev Microbiol 9: 291–306. 21383764 10.1038/nrmicro2539 PMC3611960

[6] Takeuchi O, Akira S (2010) Pattern recognition receptors and inflammation. Cell 140: 805–820. 20303872 10.1016/j.cell.2010.01.022

[7] Schroder K, Tschopp J (2010) The inflammasomes. Cell 140: 821–832. 20303873 10.1016/j.cell.2010.01.040

[8] Ashida H, Ogawa M, Kim M, Suzuki S, Sanada T, et al (2011a) *Shigella* deploy multiple countermeasures against host innate immune responses. Curr Opin Microbiol 14: 16–23. 20934372 10.1016/j.mib.2010.08.014

[9] Ashida H, Ogawa M, Mimuro H, Kobayashi T, Sanada T, et al (2011b) *Shigella* are versatile mucosal pathogens that circumvent the host innate immune system. Curr Opin Immunol 23: 448–455. 21763117 10.1016/j.coi.2011.06.001

[10] Parsot C (2009) *Shigella* type III secretion effectors: how, where, when, for what purposes? Curr Opin Microbiol 12: 110–116. 19157960 10.1016/j.mib.2008.12.002

[11] Kim DW, Lenzen G, Page AL, legrain P, Sansonetti PJ, et al (2005) The *Shigella flexneri* effector OspG interferes with innate immune responses by targeting ubiquitin-conjugating enzymes. Proc Natl Acad Sci USA 102: 14046–14051. 16162672 10.1073/pnas.0504466102 PMC1236552

[12] Sanada T, Kim M, Mimuro H, Suzuki M, Ogawa M, et al (2012) The *Shigella flexneri* effector OspI deamidates UBC13 to dampen the inflammatory response. Nature 483: 623–626. 22407319 10.1038/nature10894

[13] Newton HJ, Pearson JS, Badea L, Kelly M, Lucas M, et al (2010) The type III effectors NleE and NleB from enteropathogenic *E. coli* and OspZ from *Shigella* block nuclear translocation of NF-κB p65. PLoS Pathog 6: e1000898. 20485572 10.1371/journal.ppat.1000898 PMC2869321

[14] Haraga A, Miller SI (2003) A *Salmonella enterica* serovar Typhimurium translocated leucine-rich repeat effector protein inhibits NF-κB-dependent gene expression. Infect Immun 71: 4052–4058. 12819095 10.1128/IAI.71.7.4052-4058.2003 PMC162009

[15] Okukda J, Toyotome T, Kataoka N, Ohno M, Abe H, et al (2005) *Shigella* effector IpaH9.8 binds to a splicing factor U2AF35 to modulate host immune responses. Biochem Biophys Res Commun 333: 531–539. 15950937 10.1016/j.bbrc.2005.05.145

[16] Rohde JR, Breitkreutz A, Chenal A, Sansonetti PJ, Parsot C (2007) Type III secretion effectors of the IpaH family are E3 ubiquitin ligase. Cell Host Microbe 1: 77–83. 18005683 10.1016/j.chom.2007.02.002

[17] Ashida H, Kim M, Schmidt-Supprian M, Ma A, Ogawa M, et al (2010) A bacterial E3 ubiquitin ligase IpaH9.8 targets NEMO/IKKγ to dampen the host NF-κB-mediated inflammatory response. Nat Cell Biol 12: 66–73. 20010814 10.1038/ncb2006 PMC3107189

[18] Quezada CM, Hicks SW, Galán JE, Stebbins CE (2009) A family of *Salmonella* virulence factors functions as a distinct class of autoregulated E3 ubiquitin ligases. Proc Natl Acad Sci U S A 106: 4864–4869. 19273841 10.1073/pnas.0811058106 PMC2653562

[19] Buysse JM, Stover CK, Oaks EV, Venkatesan M, Kopecko DJ (1987) Molecular cloning of invasion plasmid antigen (*ipa*) genes from *Shigella flexneri*: analysis of *ipa* gene products and genetic mapping. J Bacteriol 169: 2561–2569. 3294797 10.1128/jb.169.6.2561-2569.1987 PMC212123

[20] Venkatesan MM, Buysse JM, Kopecko DJ (1989) Use of *Shigella flexneri ipaC* and *ipaH* gene sequences for the general identification of *Shigella* spp. and enteroinvasive *Escherichia coli* . J Clin Microbiol 27: 2687–2691. 2687318 10.1128/jcm.27.12.2687-2691.1989 PMC267109

[21] Hartman AB, Venkatesan M, Oaks EV, Buysse JM (1990) Sequence and molecular characterization of a multicopy invasion plasmid antigen gene, *ipaH*, of *Shigella flexneri* . J Bacteriol 172: 1905–1915. 1690703 10.1128/jb.172.4.1905-1915.1990 PMC208685

[22] Venkatesan MM, Buysse JM, Hartman AB (1991) Sequence variation in two *ipaH* genes of *Shigella flexneri* 5 and homology to the LRG-like family of proteins. Mol Microbiol 5: 2435–2445. 1791758 10.1111/j.1365-2958.1991.tb02089.x

[23] Hicks SW, Galán JE (2010) Hijacking the host ubiquitin pathway: structural strategies of bacterial E3 ubiquitin ligases. Curr Opin Microbiol 13: 41–46. 20036613 10.1016/j.mib.2009.11.008 PMC2822022

[24] Bernal-Bayard J, Ramos-Morales F (2009) *Salmonella* type III secretion effector SlrP is an E3 ubiquitin ligase for mammalian thioredoxin. J Biol Chem 284: 27587–27595. 19690162 10.1074/jbc.M109.010363 PMC2785687

[25] Bernal-Bayard J, Cardenal-Muñoz E, Ramos-Morales F (2010) The *Salmonella* type III secretion effector, salmonella leucine-rich repeat protein (SlrP), targets the human chaperone ERdj3. J Biol Chem 285: 16360–16368. 20335166 10.1074/jbc.M110.100669 PMC2871503

[26] Buchrieser C, Glaser P, Rusniok C, Nedjari H, D'Hauteville H, et al (2000) The virulence plasmid pWR100 and the repertoire of proteins secreted by the type III secretion apparatus of *Shigella flexneri* . Mol Microbiol 38: 760–771. 11115111 10.1046/j.1365-2958.2000.02179.x

[27] Jin Q, Yuan Z, Xu J, Wang Y, Shen Y, et al (2002) Genome sequence of *Shigella flexneri* 2a: insights into pathogenicity through comparison with genomes of *Escherichia coli* K12 and O157. Nucleic Acids Res 30: 4432–4441. 12384590 10.1093/nar/gkf566 PMC137130

[28] Ashida H, Toyotome T, Nagai T, Sasakawa C (2007) *Shigella* chromosomal IpaH proteins are secreted via the type III secretion system and act as effectors. Mol Microbiol 63: 680–693. 17214743 10.1111/j.1365-2958.2006.05547.x

[29] Toyotome T, Suzuki T, Kuwae A, Nonaka T, Fukuda H, et al (2001) *Shigella* protein IpaH_9.8_ is secreted from bacteria within mammalian cells and transported to the nucleus. J Biol Chem 276: 32071–32079. 11418613 10.1074/jbc.M101882200

[30] Bhoj VG, Chen ZJ (2009) Ubiquitylation in innate and adaptive immunity. Nature 458: 430–437. 19325622 10.1038/nature07959

[31] Hicks SW, Charron G, Hang HC, Galán JE (2011) Subcellular targeting of *Salmonella* virulence proteins by host-mediated *S*-palmitoylation. Cell Host Microbe 10: 9–20. 21767808 10.1016/j.chom.2011.06.003 PMC4326042

[32] Holden NS, Squires PE, Kaur M, Bland R, Jones CE, et al (2008) Phorbol ester-stimulated NF-κB-dependent transcription: roles for isoforms of novel protein kinase C. Cell Signal 20: 1338–1348. 18436431 10.1016/j.cellsig.2008.03.001

[33] Rosse C, Linch M, Kermorgant S, Cameron AJ, Boeckeler K, et al (2010) PKC and the control of localized signal dynamics. Nat Rev Mol Cell Biol 11: 103–112. 20094051 10.1038/nrm2847

[34] Cossart P, Sansonetti PJ (2004) Bacterial invasion: the paradigms of enteroinvasive pathogens. Science 304: 242–248. 15073367 10.1126/science.1090124

[35] Ogawa M, Handa Y, Ashida H, Suzuki M, Sasakawa C (2008) The versatility of *Shigella* effectors. Nat Rev Microbiol 6: 11–16. 18059288 10.1038/nrmicro1814

[36] Carayol N, Tran Van Nhieu G (2013) Tips and tricks about *Shigella* invasion of epithelial cells. Current Opin Microbiol 16: 1–6. 10.1016/j.mib.2012.11.010 23318141

[37] Suzuki T, Yoshikawa Y, Ashida H, Iwai H, Toyotome T, et al (2006) High vaccine efficacy against shigellosis of recombinant noninvasive *Shigella* mutant that expresses *Yersinia* invasin. J Immunol 177: 4709–4717. 16982910 10.4049/jimmunol.177.7.4709

[38] Thome M (2008) Multifunctional roles for MALT1 in T-cell activation. Nat Rev Immunol 8: 495–500. 18575460 10.1038/nri2338

[39] Blonska M, Lin X (2011) NF-κB signaling pathways regulated by CARMA family of scaffold proteins. Cell Res 21: 55–70. 21187856 10.1038/cr.2010.182 PMC3193407

[40] Oeckinghaus A, Hayden MS, Ghosh S (2011) Crosstalk in NF-κB signaling pathways. Nat Immunol 12: 695–708. 21772278 10.1038/ni.2065

[41] Li S, Wang L, Berman MA, Zhang Y, Dorf ME (2006) RNAi screen in mouse astrocytes identifies phosphatases that regulate NF-κB signaling. Mol Cell 24: 497–509. 17188031 10.1016/j.molcel.2006.10.015 PMC2572259

[42] Li S, Wang L, Dorf ME (2009) PKC phosphorylation of TRAF2 mediates IKKα/βrecruitment and K63-linked polyubiquitination. Mol Cell 33: 30–42. 19150425 10.1016/j.molcel.2008.11.023 PMC2643372

[43] Tada K, Okazaki T, Sakon S, Kobarai T, Kurosawa K, et al (2001) Critical roles of TRAF2 and TRAF5 in tumor necrosis factor-induced NF-κB activation and protection from cell death. J Biol Chem 276: 36530–36534. 11479302 10.1074/jbc.M104837200

[44] Singer AU, Rohde JR, Lam R, Skarina T, Kagan O, et al (2008) Structure of the *Shigella* T3SS effector IpaH defines a new class of E3 ubiquitin ligases. Nat Struct Mol Biol 15: 1293–1301. 18997778 10.1038/nsmb.1511 PMC2764551

[45] Zhu Y, Li H, Hu L, Wang J, Zhou Y, et al (2008) Structure of a *Shigella* effector reveals a new class of ubiquitin ligases. Nat Struct Mol Biol 15: 1302–1308. 18997779 10.1038/nsmb.1517

[46] Dupont N, Lacas-Gervais S, Bertout J, Paz I, Freche B, et al (2009) *Shigella* phagocytic vacuolar membrane remnants participate in the cellular response to pathogen invasion and are regulated by autophagy. Cell Host Microbe 6: 137–149. 19683680 10.1016/j.chom.2009.07.005

[47] Shahnazari S, Yen WL, Birmingham CL, Shiu J, Namolovan A, et al (2011) A diacylglycerol-dependent signaling pathway contributes to regulation of antibacterial autophagy. Cell Host Microbe 8: 137–146. 10.1016/j.chom.2010.07.002 PMC3668700 20674539

[48] Tattoli I, Sorbara MT, Vuckovic D, Ling A, Soares F, et al (2012) Amino acid starvation induced by invasive bacterial pathogens triggers an innate host defense program. Cell Host Microbe 11: 563–575. 22704617 10.1016/j.chom.2012.04.012

[49] Shaughnessy LM, Lipp P, Lee KD, Swanson JA (2007) Localization of protein kinase C ε to macrophage vacuoles perforated by *Listeria monocytogenes* cytolysin. Cell Microbiol 9: 1695–1704. 17346313 10.1111/j.1462-5822.2007.00903.x PMC1974810

[50] Hayden MS, Ghosh S (2008) Shared principles in NF-κB signaling. Cell 132: 344–362. 18267068 10.1016/j.cell.2008.01.020

[51] Hasegawa M, Fujimoto Y, Lucas PC, Nakano H, Fukase K, et al (2008) A critical role of RICK/RIP2 polyubiquitination in Nod-induced NF-κB activation. EMBO J 27: 373–383. 18079694 10.1038/sj.emboj.7601962 PMC2234345

[52] Blackwell K, Zhang L, Thomas GS, Sun S, Nakano H, et al (2009) TRAF2 phosphorylation modulates tumor necrosis factor alpha-induced gene expression and cell resistance to apoptosis. Mol Cell Biol 29: 303–314. 18981220 10.1128/MCB.00699-08 PMC2612514

[53] Thomas GS, Zhang L, Blackwell K, Habelhah H (2009) Phosphorylation of TRAF2 within its RING domain inhibits stress-induced cell death by promoting IKK and suppressing JNK activation. Cancer Res 69: 3665–36672. 19336568 10.1158/0008-5472.CAN-08-4867 PMC2669835

[54] Zhang L, Blackwell K, Altaeva A, Shi Z, Habelhah H (2011) TRAF2 phosphorylation promotes NF-κB-dependent gene expression and inhibits oxidative stress-induced cell death. Mol Biol Cell 22: 128–140. 21119000 10.1091/mbc.E10-06-0556 PMC3016971

[55] Girardin SE, Boneca IG, Carneiro LA, Antignac A, Jéhanno M, et al (2003) Nod1 detects a unique muropeptide from gram-negative bacterial peptidoglycan. Science 300: 1584–1587. 12791997 10.1126/science.1084677

[56] Kufer TA, Kremmer E, Adam AC, Philpott DJ, Sansonetti PJ (2007) The pattern-recognition molecule Nod1 is localized at the plasma membrane at sites of bacterial interaction. Cell Microbiol 10: 477–486. 17970764 10.1111/j.1462-5822.2007.01062.x

[57] Travassos LH, Carneiro LA, Ramjeet M, Hussey S, Kim YG, et al (2010) Nod1 and Nod2 direct autophagy by recruiting ATG16L1 to the plasma membrane at the site of bacterial entry. Nat Immunol 11: 55–62. 19898471 10.1038/ni.1823

[58] Watarai M, Kamata Y, Kozaki S, Sasakawa C (1997) rho, a small GTP-binding protein, is essential for *Shigella* invasion of epithelial cells. J Exp Med 185: 281–292. 9016877 10.1084/jem.185.2.281 PMC2196126

